# Effect of socioeconomic inequalities on cholecystectomy outcomes: a 10-year population-based analysis

**DOI:** 10.1186/s12939-018-0739-7

**Published:** 2018-02-13

**Authors:** Ping Lu, Nan-Ping Yang, Nien-Tzu Chang, K. Robert Lai, Kai-Biao Lin, Chien-Lung Chan

**Affiliations:** 1 School of Economics and Management, Xiamen University of Technology, Xiamen, 361024 China; 2 Department of Information Management, Yuan Ze University, Taoyuan, 32003 Taiwan; 3 Department of Surgery, Keelung Hospital, Ministry of Health and Welfare, Keelung, 20148 Taiwan; 4 Institute of Public Health, National Yang-Ming University, Taipei, 11221 Taiwan; 5 School of Nursing, College of Medicine, National Taiwan University, Taipei, 10051 Taiwan; 6 Department of Computer Science and Engineering, Yuan Ze University, Taoyuan, 32003 Taiwan; 7 School of Computer & Information Engineering, Xiamen University of Technology, Xiamen, 361024 China; 8 Innovation Center for Big Data and Digital Convergence, Yuan Ze University, Taoyuan, 32003 Taiwan

**Keywords:** Socioeconomic status, Cholecystectomy, Gallbladder disease, Acute cholecystitis

## Abstract

**Background:**

Although numerous epidemiological studies on cholecystectomy have been conducted worldwide, only a few have considered the effect of socioeconomic inequalities on cholecystectomy outcomes. Specifically, few studies have focused on the low-income population (LIP).

**Methods:**

A nationwide prospective study based on the Taiwan National Health Insurance dataset was conducted during 2003–2012. The International Classification of ICD-9-CM procedure codes 51.2 and 51.21–51.24 were identified as the inclusion criteria for cholecystectomy. Temporal trends were analyzed using a joinpoint regression, and the hierarchical linear modeling (HLM) method was used as an analytical strategy to evaluate the group-level and individual-level factors. Interactions between age, gender and SES were also tested in HLM model.

**Results:**

Analyses were conducted on 225,558 patients. The incidence rates were 167.81 (95% CI: 159.78–175.83) per 100,000 individuals per year for the LIP and 123.24 (95% CI: 116.37–130.12) per 100,000 individuals per year for the general population (GP). After cholecystectomy, LIP patients showed higher rates of 30-day mortality, in-hospital complications, and readmission for complications, but a lower rate of routine discharge than GP patients. The hospital costs and length of stay for LIP patients were higher than those for GP patients. The multilevel analysis using HLM revealed that adverse socioeconomic status significantly negatively affects the outcomes of patients undergoing cholecystectomy. Additionally, male sex, advanced age, and high Charlson Comorbidity Index (CCI) scores were associated with higher rates of in-hospital complications and 30-day mortality. We also observed that the 30-day mortality rates for patients who underwent cholecystectomy in regional hospitals and district hospitals were significantly higher than those of patients receiving care in a medical center.

**Conclusion:**

Patients with a disadvantaged finance status appeared to be more vulnerable to cholecystectomy surgery. This result suggested that further interventions in the health care system are necessary to reduce this disparity.

## Background

Gallbladder disease remains one of the most common problems encountered in surgical intervention and, if managed incorrectly, can lead to high rates of morbidity and mortality [1]. Approximately 20 million people in the USA have gallstones, leading to over one million hospitalizations and 700,000 operative procedures annually [2, 3]. The overall prevalence of gallstone disease is 5.0% (4.6% in men, 5.4% in women) with no significant sex differences in Taiwan [4]. The hospital admission rate for severe gallstone disease in Taiwan increases with advancing age, and the age-standardized rate (95% CI) per 1000 population is 0.60 (0.59–0.60) for men and 0.59 (0.59–0.60) for women [5]. Symptomatic gallstones are treated by surgically removing the gallbladder, and cholecystectomy is largely accepted as the standard procedure for treating benign gallbladder disease [6, 7]. Although numerous epidemiological studies on cholecystectomy have been conducted worldwide, few studies have considered the effect of socioeconomic inequalities on cholecystectomy outcomes. To the best of our knowledge, relatively few studies have focused on the low-income population (LIP) in Asian settings.

In some Western countries, socioeconomic status (SES) has been reported to have a strong association with postoperative mortality in numerous studies [8–10]. For example, Andrew et al. [11] suggested that environmental factors associated with SES, such as level of education, income, occupational status, and neighborhood, play a role in gallstone pathogenesis. Vishnu et al. [12] reported that patients with Medicaid and lower SES had poorer outcomes after cholecystectomy. However, other researchers found no relationship between socioeconomic conditions and the prevalence of cholelithiasis [13, 14]. For example, Carbonell et al. [14] performed a nationwide study of 93,758 patients and demonstrated that income, insurance status, and race did not play a role in morbidity or mortality for patients who underwent cholecystectomy; moreover, academic or teaching status at the hospital did not influence patient outcomes. Therefore, previous studies have yielded conflicting results on the effects of socioeconomic inequalities on cholecystectomy outcomes. It is important to recognize patient populations that might be at a higher risk of mortality and postoperative complications to identify potentially modifiable risk factors [12]. Thus, in-depth population-based studies and analyses on the effects of socioeconomic inequalities on cholecystectomy must be conducted. The main purpose of this population-based study was to investigate the effect of socioeconomic inequalities on cholecystectomy outcomes in Taiwan. We also compared the temporal trends of cholecystectomy between the LIP and GP using a joinpoint regression and assessed the odds ratios of in-hospital complications and 30-day mortality between the LIP and GP for all enrolled patients using a multilevel analysis with HLM. We expected to be able to provide some valuable information to assist surgeons in decision-making and to make recommendations to health policy decision-makers with respect to the development of preventive strategies.

## Methods

### Study subjects and data source

We retrieved the inpatient data from Taiwan’s National Health Insurance Research Database (NHIRD), which is a nationwide medical claims database for Taiwan’s National Health Insurance (NHI) program. This program launched in 1995 and reached a 99.9% coverage rate by 2011. Various data subsets, such as inpatient expenditures, details of prescription orders, and clinics or ambulatory care expenditures, are included in the NHIRD. To protect privacy, all the subjects’ ID numbers were double encrypted. All researchers were properly trained and were required to declare and sign written agreement before using these data subsets.

### Data definition

To examine the effects of socioeconomic inequalities on cholecystectomy outcomes in Taiwan, we used the diagnostic codes of the International Classification of Diseases, Ninth Revision, Clinical Modification (ICD-9-CM). Cholecystectomy was identified by ICD-9-CM procedure codes 51.2 and 51.21–51.24, and percutaneous cholecystostomy (PC) was identified as ICD-9-CM procedure code 51.01 [15]. To avoid the effects of PC on the outcome of patients who underwent cholecystectomy, we excluded patients who had initially undergone PC before cholecystectomy during a prior hospitalization. However, when patients had undergone both PC and cholecystectomy during the same hospitalization, they were classified as cholecystectomy patients. Only patients 18 years or older who had undergone cholecystectomy were included. To analyze the procedure causes, acute cholecystitis (AC) with a calculus/stone was defined as patients with ICD-9-CM diagnosis codes 574.0, 574.3, and 574.6; AC without a calculus/stone was defined as patients with the ICD-9-CM diagnosis code 575.0; calculus without AC referred to patients with ICD-9-CM diagnosis codes 574.1, 574.2, 574.4, 574.5, 574.7, 574.8, or 574.9; other disorders of the gallbladder or biliary tract were defined as patients with ICD-9-CM diagnosis codes 575 or 576, excluding diagnosis code 575.0; and malignant neoplasms of digestive organs and the peritoneum included patients with ICD-9-CM diagnosis codes 150–159, excluding diagnosis codes 574, 575, and 576.

### Socioeconomic status

To evaluate the effects of SES, the enrolled subjects were classified into the LIP and the general population (GP) according to whether the subjects met the criteria of Taiwan’s Social Assistance Act and was registered in Taiwan’s NHI database. For easy recognition, Taiwan’s NHI database marked the LIP group as the fifth class beneficiary [16]. The GP refers to individuals who are not classified as LIP.

### Outcome measurements

#### Length of hospital stays (LOS)

The period between admission and discharge was defined as the LOS (measured in days). The LOS was measured as 1 day for patients discharged on the same day that they were admitted to the hospital [17].

#### Hospital costs

Hospital costs were calculated by summing all items enumerated in the hospital discharge summary, including operation-associated costs and ward costs. Operation-associated costs included anesthesia and surgery fees and the costs of medical supplies used during an operation. Surplus costs were classified as ward costs. Costs are expressed in U.S. dollars (USD). In 2007, one USD dollar was equivalent to approximately 32.64 Taiwan dollars [17].

#### In-hospital complications

We examined all-cause, nonfatal in-hospital morbidity rates based on ICD-9-CM codes. Complications were grouped into 9 categories (mechanical wound complications, infections, urinary complications, pulmonary complications, systemic complications, complications arising during procedures, specific complications of the gallbladder/digestive system, respiratory complications, and others). Because the NHIRD inpatient dataset includes inpatient data only, complications occurring after hospital discharge were not considered in our analysis.

#### 30-day mortality and in-hospital mortality

Thirty-day mortality was used to refer to patients who died within one month after undergoing cholecystectomy; this variable also included patients who died during hospitalization. In-hospital mortality was used to refer to patients undergoing cholecystectomy who died during hospitalization.

#### Rate of routine discharge

The NHIRD provides information on patient discharge statuses (1, treated and discharged; 2, continued to hospital; 3, transferred to outpatient treatment; 4, death; 5, discharge against medical advice; 6, referral; 7, change in status; 8, abscond; 9, suicide; 0, other; and A, discharged while dying). Patients were grouped into the following categories: routine discharge (1, 3) and non-routine discharge (0, 2, 4–9, and A).

#### Readmission due to complications

Readmission due to complications was designated when readmission occurred due to the diagnosis of a commonly encountered postoperative complication listed in Appendix within 1 month after the cholecystectomy.

### Statistical analysis

Descriptive statistics for comparing baseline characteristics were determined based on the number of cases, percentages, and 95% confidence intervals (CIs) for the estimated rates. Chi-square tests and independent-t tests were used to evaluate the significance of frequency and continuous variable differences between two subgroups, respectively. Statistical significance was set at 0.05. Temporal trends were analyzed using a joinpoint regression, which is a statistical method that fits a series of joined straight lines between statistically significant changes in trends (joinpoints). We estimated the change between joinpoints using the National Cancer Institute’s Joinpoint Regression Program Version 4.3.1.0 [18, 19]. Long-term trends over the entire time series were designated average annual percentage changes (AAPCs) and were estimated as the weighted average of short-term annual percentage changes with weights equal to the length of the short-term line segment [20].

To evaluate the risk factors for 30-day mortality and in-hospital complications after cholecystectomy, a multiple logistic regression analysis was performed, and the adjusted odds ratio (AOR) was calculated. A multilevel analysis (or the hierarchical linear modeling (HLM) method) was used as an analytical strategy, which allowed the evaluation of group-level and individual-level factors [21]. Interactions between age, gender and SES were also tested in the HLM model. The hypothesis and formulas of the HLM analysis used in the present study were as follows.

Level 1 HLM model

1 $$ {\displaystyle \begin{array}{l} Yij=\beta 0+\beta 1\times (gender)+\beta 2\times \left(\mathrm{a} ge\; group\;1\right)+\beta 3\times \left( age\; group\;2\right)\\ {}+\beta 4\times \left( age\; group\;3\right)+\beta 5\times \left( age\; group\;4\right)+\beta 6\times \left( age\; group\;5\right)\\ {}+\beta 7\times \left( CCI\;1\right)+\beta 8\times \left( CCI\;2\right)+\beta 9\times \left( CCI\;3\right)\beta +\beta 10\times \left( AC\; without\;a\;C/S\right)+\beta 11\times \left( Calculus\kern0.17em without\; AC\right)+\beta 12\times (ODGBT)+\beta 13\times (MNDOP)+\beta 14\times (Others)+\gamma .\end{array}} $$

Level 2 HLM model

2 $$ {\displaystyle \begin{array}{l}\beta 0=\gamma 00+\gamma 01\times (SES)+\gamma 02\times \left( regional\kern0.5em hospital\right)+\gamma 03\times \left( district\kern0.5em hospital\right)\\ {}+\gamma 03\times \left({\mathrm{SES}}^{\ast}\kern0.5em gender\right)+\gamma 03\times \left({\mathrm{SES}}^{\ast}\kern0.5em age\kern0.5em group\kern0.5em 3\right)+\gamma 03\times \left({\mathrm{SES}}^{\ast}\kern0.5em age\kern0.5em group\kern0.5em 4\right)\\ {}+\gamma 03\times \left({\mathrm{SES}}^{\ast}\kern0.5em age\kern0.5em group\kern0.5em 5\right)+\mu 0.\end{array}} $$

All statistical analyses were performed using the Statistical Package for Social Sciences for Windows (SPSS for Windows Version 22.0).

## Results

Of the 225,558 sampled patients who underwent cholecystectomy during 2003–2012, the incidence rate was 167.81 (95% CI: 159.78–175.83) per 100,000 individuals per year for the LIP and 123.24 (95% CI: 116.37–130.12) per 100,000 individuals per year for the GP. The mean ages were 54.9 ± 16.7 years (±standard deviation) and 56.8 ± 16.2 years for LIP and GP patients, respectively. Cholecystectomy was performed significantly more often and in slightly younger patients in the LIP than in the GP.

**Fig. 1:**
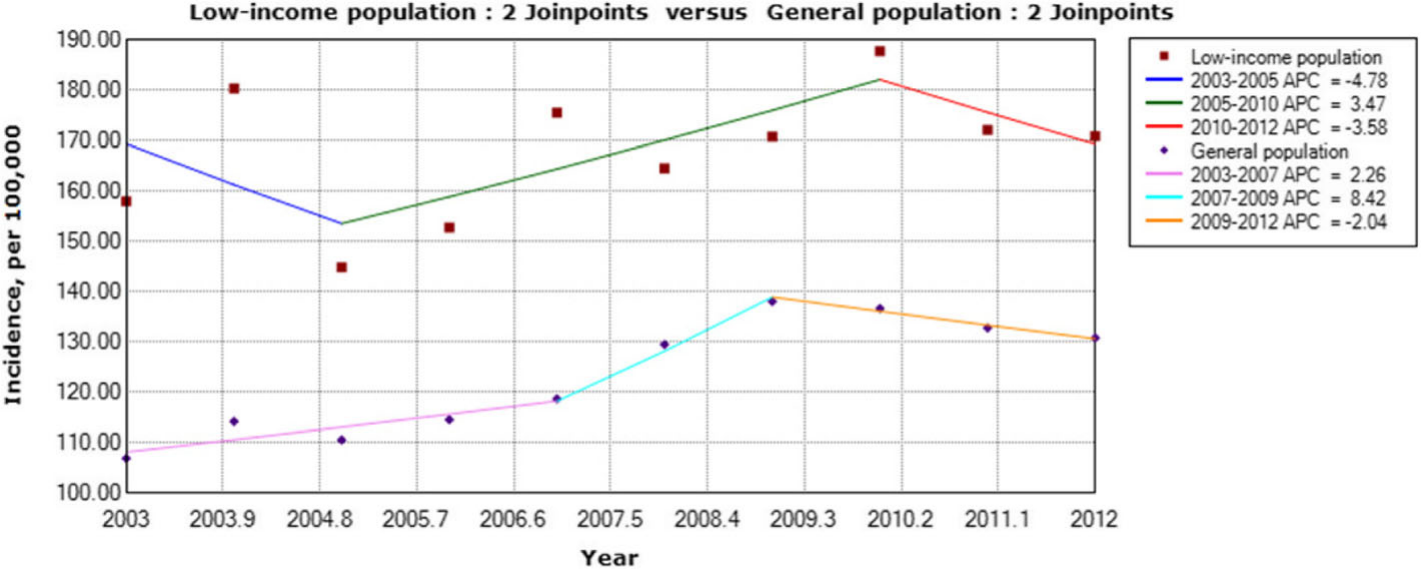
Comparison of cholecystectomy incidence in the LIP and GP in Taiwan

Based on multiple permutation tests that kept the overall level of type I errors to less than 0.05, the final selected model detected 2 joinpoints in 2005 and 2010 for LIP patients and 2 joinpoints in 2007 and 2009 for GP patients (Fig. 1). During the entire retrospective period, the incidence for the GP increased slowly (AAPC = 2.1%) from 106.86 to 130.79 per 100,000 individuals. Meanwhile, the incidence for the LIP remained constant at approximately 170.00 per 100,000 individuals (AAPC = 0%). The short-term trends (APCs) between LIP and GP patients were different, although similar trends were observed after 2004.

As shown in Table 1, the differences in gender, age stratum, Charlson Comorbidity Index (CCI) score, cause of procedure and hospital level were significant between the LIP and GP (*p* < 0.05). Male and female patients were essentially evenly distributed in both the LIP and GP. In the LIP, there was a higher proportion of young patients (aged 30–39 and aged 40–49) and a lower proportion of elderly patients (aged over 50) than in the GP. Patients aged 40–49 years comprised the highest proportion (27.79%) of LIP patients, whereas patients aged over 70 years comprised the highest proportion (25.32%) of GP patients (Table 1).

**Table 1:** Demographic characteristic of patients undergoing cholecystectomy in Taiwan, 2003–2012

Variables	Low-income population (*n* = 2454)		General population (*n* = 223,104)		*p*-value
	Total no.	Percent (%)	Total no.	Percent (%)	
Gender					0.028
Female	1192	48.57%	113,346	50.80%	
Male	1262	51.43%	109,758	49.20%	
Age stratum					< 0.001
18–29 y/o	79	3.22%	10,438	4.68%	
30–39 y/o	353	14.38%	26,965	12.09%	
40–49 y/o	682	27.79%	37,976	17.02%	
50–59 y/o	428	17.44%	48,679	21.82%	
60–69 y/o	312	12.71%	42,557	19.07%	
70 y/o or more	600	24.45%	56,489	25.32%	
CCI score					< 0.001
0	1711	69.72%	155,823	69.84%	
1	405	16.50%	28,752	12.89%	
2	185	7.54%	21,703	9.73%	
≥ 3	153	6.23%	16,826	7.54%	
Cause of procedure					< 0.001
AC with a C/S	786	32.03%	63,605	28.51%	
AC without a C/S	154	6.28%	9019	4.04%	
Calculus without AC	1216	49.55%	113,825	51.02%	
ODGBT	131	5.34%	13,521	6.06%	
MNDOP	104	4.24%	18,289	8.20%	
Others	63	2.57%	4845	2.17%	
Hospital level					< 0.001
Medical center	730	29.75%	105,542	47.31%	
Regional hospital	1345	54.81%	99,729	44.70%	
District hospital	379	15.44%	17,833	7.99%	

AC with a C/S: Acute cholecystitis with a calculus/stone

AC without a C/S: Acute cholecystitis without a calculus/stone

Calculus without AC: Calculus without acute cholecystitis

ODGBT: Other disorders of the gallbladder or biliary tract

MNDOP: Malignant neoplasm of digestive organs and peritoneum

CCI: Charlson Comorbidity Index

The primary reason for a cholecystectomy was a calculus without AC (LIP: 49.55%, GP: 51.02%), followed by AC with a calculus/stone (LIP: 32.03%, GP: 28.51%) for both the LIP and GP. The proportion of malignant neoplasms of digestive organs and peritoneum (MNDOP) was nearly twice as high in the GP as in the LIP. Nearly 30% of patients who underwent cholecystectomy had CCI scores of 1 or more for both the LIP and the GP (LIP: 30.28%, GP 30.16%). Unlike the LIP, GP patients preferred to undergo cholecystectomy at medical centers and were less likely to choose regional or district hospitals.

The overall 30-day mortality for patients who underwent cholecystectomy was 2.21%. LIP patients showed higher rates of 30-day mortality (LIP: 4.65% vs. GP: 2.18%, *p* < 0.001), in-hospital complications (LIP: 5.62% vs. GP: 4.01%, *p* = 0.008), and readmission for complications (LIP: 1.83% vs. GP: 1.09%, *p* < 0.001) but showed a lower rate of routine discharge (LIP: 94.91% vs. GP: 97.67%, *p* < 0.001) than GP patients (Table 2).

**Table 2:** Characteristics of 30-day mortality, in-hospital complications, rate of routine discharge and readmission for complications, and length of hospital stay

Variables	All No. (%)	Low-income population No. (%), mean ± SE	General population No. (%), mean ± SE	*p*-value^c^
30-day mortality	4987 (2.21%)	114 (4.65%)	4873 (2.18%)	< 0.001
In-hospital mortality	3602 (1.60%)	81 (3.30%)	3521 (1.58%)	< 0.001
In-hospital complications^a^	9089 (4.03%)	138 (5.62%)	8951 (4.01%)	0.008
Specific complications of the gallbladder/digestive system	4674 (2.07%)	62 (2.53%)	4612 (2.07%)	0.112
Infections	2850 (1.26%)	53 (2.16%)	2797 (1.25%)	< 0.001
Mechanical wound complications	1320 (0.59%)	20 (0.81%)	1300 (0.58%)	0.133
Complications during procedure	882 (0.39%)	15 (0.61%)	867 (0.39%)	0.079
Pulmonary complications	310 (0.14%)	5 (0.20%)	305 (0.14%)	0.373
Systemic complications	156 (0.07%)	3 (0.12%)	153 (0.07%)	0.314
Respiratory complications	74 (0.03%)	1 (0.04%)	73 (0.03%)	0.827
Urinary complications	22 (0.01%)	1 (0.04%)	21 (0.01%)	0.118
Other	40 (0.02%)	1 (0.04%)	39 (0.02%)	0.389
Rate of routine discharge	220,244 (97.64%)	2329 (94.91%)	217,915 (97.67%)	< 0.001
Treatment and discharge	12,469 (5.53%)	94 (3.83%)	12,375 (5.55%)	< 0.001
Transferred to outpatient treatment	207,775 (92.12%)	2235 (91.08%)	205,540(92.13%)	0.055
Readmission for complications^b^	2475 (1.10%)	45 (1.83%)	2430 (1.09%)	< 0.001
Length of hospital stay	–	12.13 ± 0.03	9.49 ± 0.02	< 0.001
Hospital costs	–	3263.82 ± 65.89	2832.17 ± 6.00	< 0.001

^a^Two or more complications occurred for the same patient; therefore, the total number of patients with complications was less than the sum of the number of patients with each independent complication

^b^Readmission for complications was defined as readmission with the diagnosis of a commonly encountered postoperative complication within 1 month after the cholecystectomy

^c^*p*-values for length of hospital stay and hospital costs were analyzed with independent t-tests; other variables were analyzed with Chi-square tests

**Fig. 2:**
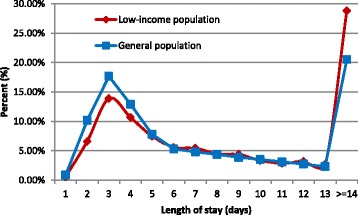
Frequency distributions of length of hospital stay for patients who underwent cholecystectomy

**Fig. 3:**
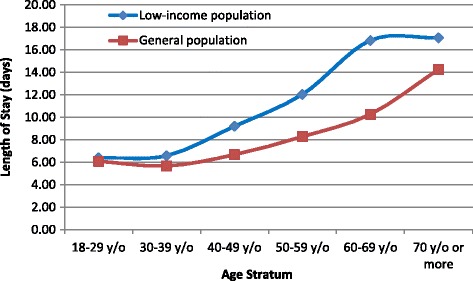
Mean length of hospital stay for patients who underwent cholecystectomy according to age group

The mean LOS for LIP patients was significantly longer than that of GP patients (12.13 ± 0.28 vs. 9.49 ± 0.02, *p* < 0.001). Correspondingly, the mean cost for LIP patients was much higher than that for GP patients (3263.82 ± 65.89 vs. 2832.17 ± 6.00 USD, *p* < 0.001) (Table 2). The detailed LOS distribution for the GP and LIP is shown in Fig. 2. More GP patients were hospitalized for 1–6 days, whereas more LIP patients were hospitalized for over 14 days. Figure 3 illustrates that the mean LOS increased with advancing age for both LIP and GP patients, and the mean LOS was higher in LIP patients than in GP patients for every age group.

A multilevel analysis with HLM was used to evaluate the individual effects (i.e., gender, age, cause of procedure, CCI Score) and the group effects (i.e., SES and hospital level) on in-hospital complications and 30-day mortality. As shown in Table 3, male patients showed higher rates of 30-day mortality (AOR = 1.341, *p* < 0.001) and in-hospital complications (AOR = 1.470, *p* < 0.001). Compared to the 18-to-29-year-olds, patients aged 30–39 exhibited a lower rate of in-hospital complications (AOR = 0.842, *p* = 0.026). However, patients aged 40–49 showed a higher rate of 30-day mortality (AOR = 1.410, *p* = 0.013), and patients aged 60–69 and 70 years or older displayed much higher rates of both in-hospital complications and 30-day mortality. In particular, the AORs of patients aged 70 years or older were 2.369 for in-hospital complications and 7.831 for 30-day mortality. In general, aging was associated with a higher risk of complications and mortality. Additionally, different reasons for the procedure showed significantly different complications and mortality risks compared to cases of AC with a calculus/stone. In particular, patients with the cause ‘other’ had a significantly higher risk of 30-day mortality (AOR = 9.799, *p* < 0.001). Lower hospital levels were associated with a higher risk of 30-day mortality, and an adverse trend was also reported for in-hospital complications. More importantly, the LIP patients faced clearly higher rates of 30-day mortality (AOR = 2.197, *p* < 0.001) and increased in-hospital complications (AOR = 1.599, *p* < 0.001). The only significant interaction factor was SES*70 y/o or more, which was identified as a protective factor associated with reduced in-hospital complications. No significant interaction terms between age, gender and SES were identified for all other factors in the HLM analysis.

**Table 3:** Multilevel analysis (with HLM) of the risk factors for in-hospital mortality and 30-day mortality among patients who underwent cholecystectomy in Taiwan, 2003–2012

Variable	In-hospital complications			30-day mortality		
	β-value	AOR	*p-*value	β-value	AOR	*p-*value
Gender^a^						
Female^c^		1.0			1.0	
Male	0.381	1.464 (1.399, 1.532)	< 0.001	0.290	1.337 (1.257, 1.422)	< 0.001
Age stratum^a^						
18–29 y/o^c^		1.0			1.0	
30–39 y/o	−0.173	0.841 (0.722, 0.980)	0.025	0.030	1.030 (0.767, 1.385)	0.843
40–49 y/o	−0.017	0.983 (0.852, 1.133)	0.808	0.335	1.398 (1.066, 1.835)	0.016
50–59 y/o	0.276	1.318 (1.149, 1.511)	< 0.001	0.524	1.689 (1.298, 2.198)	< 0.001
60–69 y/o	0.506	1.658 (1.446, 1.902)	< 0.001	1.045	2.842 (2.192, 3.687)	< 0.001
70 y/o or more	0.869	2.383 (2.085, 2.725)	< 0.001	2.065	7.884 (6.111, 10.171)	< 0.001
CCI score^a^						
0^c^		1.0			1.0	
1	0.064	1.067 (0.996, 1.142)	0.065	0.142	1.152 (1.052, 1.261)	0.003
2	0.378	1.459 (1.354, 1.572)	< 0.001	0.848	2.336 (2.119, 2.575)	< 0.001
≥ 3	0.604	1.829 (1.694, 1.974)	< 0.001	1.343	3.831 (3.488, 4.207)	< 0.001
Cause of procedure^a^						
AC with a C/S^c^		1.0			1.0	
AC without a C/S	0.247	1.280 (1.151, 1.423)	< 0.001	0.975	2.650 (2.374, 2.958)	< 0.001
Calculus without AC	−0.122	0.885 (0.836, 0.937)	< 0.001	−0.178	0.837 (0.772, 0.908)	< 0.001
ODGBT	1.019	2.770 (2.562, 2.995)	< 0.001	0.458	1.581 (1.394, 1.792)	< 0.001
MNDOP	0.389	1.476 (1.348, 1.615)	< 0.001	0.379	1.461 (1.303, 1.637)	< 0.001
Others	0.988	2.686 (2.393, 3.015)	< 0.001	2.282	9.793 (8.625, 11.120)	< 0.001
Hospital level^b^						
Medical center^c^		1.0			1.0	
Regional hospital	−0.108	0.898 (0.857, 0.940)	< 0.001	0.551	1.734 (1.624, 1.852)	< 0.001
District hospital	−0.228	0.796 (0.724, 0.874)	< 0.001	0.950	2.585 (2.335, 2.861)	< 0.001
SES^b^						
General population^c^		1.0			1.0	
Low-income population	0.430	1.537 (1.039, 2.274)	0.023	0.910	2.484 (1.543, 4.001)	< 0.001
SES*Gender^b^	0.400	1.492 (0.993, 2.240)	0.058	0.187	1.205 (0.773, 1.879)	0.410
SES*50–59 y/o^b^	−0.217	0.805 (0.474, 1.367)	0.421	−0.167	0.846 (0.424, 1.689)	0.635
SES*60–69 y/o^b^	−0.073	0.930 (0.547, 1.582)	0.785	−0.224	0.800 (0.400, 1.598)	0.526
SES*70 y/o or more^b^	−0.552	0.576 (0.364, 0.910)	0.020	−0.384	0.681 (0.409, 1.135)	0.141

^a^Individual level. ^b^Cluster level. ^c^Reference group. AOR: adjusted odds ratio. SES: Socioeconomic status

## Discussion

In Taiwan, low-income households are defined as those with an average per-person gross monthly income of less than the monthly minimum living expense standard of that residence region. The minimum living expense standard is defined as 60% of the average monthly disposable income for each region. In addition, family property is not permitted to exceed a certain amount, as determined by the central or municipal authorities in the corresponding year [22]. The Taiwan Ministry of the Interior has conducted six surveys regarding living conditions in the LIP and moderate LIP. The first survey was conducted in 1990, and the latest was performed in 2013 [23]. The survey results showed that long-term illness is the second leading cause of people becoming poor (the first leading cause is all family members being unable to work), and the LIP was more subject to serious diseases than the GP in Taiwan [24–26]. Hence, conducting in-depth research and analyses of the effects of socioeconomic inequalities on cholecystectomy in LIP patients is necessary; such studies may lead to treatment suggestions for medical research institutions and may assist surgeons in making decisions concerning the management of LIP patients with gallbladder disease and the judicious use of cholecystectomy.

In our analysis, we found that the overall incidence of cholecystectomy in the LIP was 36.17% higher than that in the GP. Thus, the risk of gallbladder disease in the LIP was higher than that in the GP. Moreover, LIP patients differed significantly from GP patients in hospital choice; they were more likely to undergo cholecystectomy in regional or district hospitals, which accounted for 70.25% of their total procedures. However, nearly half of the GP patients underwent cholecystectomy in medical centers (Table 1). Therefore, LIP patients are at a disadvantage in accessing medical resources compared to GP patients; they tend to live in more remote areas than GP patients, and they may need to travel further than GP patients to obtain premium medical care.

As shown in Table 2, LIP patients showed higher rates of 30-day-mortality, in-hospital complications and readmission for complications, but they had a slightly lower rate of routine discharge than GP patients. Thus, the overall situation after surgery is worse for LIP patients than for GP patients. In addition, the hospital costs and LOS for the LIP patients were higher than those of the GP patients (Table 2), which occurred primarily because more LIP patients were hospitalized for over 14 days compared to GP patients (Fig. 2). Furthermore, LIP patients constitute a more vulnerable population than GP patients.

A multilevel analysis using HLM was performed with data from all enrolled patients to assess the odds ratios of in-hospital complications and 30-day mortality. As shown in Table 3, male sex, elderly patients, and CCI score were associated with mortality, which has been reported in previous studies [12, 27]. Notably, 30-day mortality differed greatly in patient groups separated by reason for the procedure. When we used the patient group that underwent cholecystectomy because of ‘AC with a calculus/stone’ as a reference group and excluded the patient group requiring cholecystectomy because of ‘calculus without AC’, the 30-day mortality of the remaining patient groups was significantly higher than that of the reference group. For procedures classified as ‘other’, such as uncommon gallbladder-related diseases or digestive-related diseases, the 30-day mortality was much higher than that of other diseases (AOR = 9.799, *p* < 0.001). Common sense dictates that more serious diseases exhibit increased mortality rates. However, the mortality increased dramatically when the cause of the procedure was ‘other’, and this finding should be taken more seriously when making surgical decisions. Surprisingly, the interaction term “SES*70 y/o or more” was identified as a protective factor of reduced in-hospital complications. More investigations are needed to analyze the possible cause, such as a similar healthy worker effect (HWE) [28] and/or other effects.

In addition, our analysis found that the 30-day cholecystectomy mortality in regional hospitals was higher than that in medical centers, and regional hospitals also showed higher mortality rates than district hospitals. Thus, fewer hospital resources lead to higher mortality rates. Nevertheless, as previously mentioned, the LIP has poorer access to health care, making them more prone to requiring surgery for an acute process such as cholecystectomy in a regional or district hospital; poorer access is also responsible for higher mortality in LIP patients than in GP patients. Therefore, LIP patients requiring surgery should select better hospitals to the best of their abilities, particularly for serious illnesses. Meanwhile, the government must balance medical resources, enhance the overall quality of medical care in remote areas, and improve patient care for the LIP. Finally, we found that the 30-day mortality of LIP patients was higher than that of GP patients (AOR = 2.197, *p* < 0.001, Table 3), which confirms that socioeconomic inequalities are a risk factor for cholecystectomy outcomes.

Similar to the results of 30-day mortality, male sex, elderly patients, and CCI score were associated with the rate of in-hospital complications. In addition, the rate of in-hospital complications also differed greatly in different patient groups divided by different causes of procedure. Interestingly, the lower the hospital level patients were admitted to, the lower the risk of in-hospital complications, indicating a reversal of the association reported for 30-day mortality; however, additional research is necessary to confirm this relationship. We also observed that the rate of in-hospital complications in LIP patients was higher than that in GP patients (AOR = 1.599, *p* < 0.001, Table 3), which is consistent with the 30-day mortality association.

A huge difficulty of socioeconomic inequality studies is how to reasonably distinguish the LIP group and the GP group. A key strength of our current study is that the government of Taiwan has strict standards and procedures for the identification of low-income households, and there are special departments and staff in charge of these works. Thus, the quality of the current data can be deemed reliable. Meanwhile, this study was based on a population-based database, which allowed us to study the LIP with large sample sizes. However, the present data also have limitations. First, as in many national-level databases, we lack detailed clinical data and examination information. Second, data on postoperative conditions were not included. Third, we could not review individual medical records to ensure that the records were coded precisely; deviations may exist between the codes and the actual severity of disease conditions. Even so, this national population-based claims database can be recognized as reliable because it has been adopted in many research fields and numerous high-impact publications.

## Conclusions

In conclusion, our study confirms that socioeconomic inequalities have a negative effect on the outcomes of patients undergoing cholecystectomy. Compared to GP patients, LIP patients show higher rates of 30-day mortality, in-hospital complications, and readmission for complications, as well as a slightly lower rate of routine discharge. In addition to the factors of male sex, advanced age, CCI score, cause of procedure, and hospital level, among others, adverse SES plays an important role in the risk of mortality and complications for patients who undergo cholecystectomy. We recommend surgeons fully consider a patient’s SES and other features when performing cholecystectomy to design adequate plans to reduce the rates of mortality and complications for LIP patients. In addition, governments should balance medical resources, enhance the overall quality of medical care in remote areas, and limit disparities in access to health care for LIP patients.

## Appendix

**Table 4:** ICD-9 codes for postoperative in-hospital complications

Complications	ICD-9 codes
Mechanical wound complications	998.12, 998.13, 998.3, 998.6, and 998.83
Infections	998.5, 998.51, and 998.59
Urinary complications	997.5
Pulmonary complications	512.1, 518.4, 518.5, and 997.3
Systemic complications	998.0 and 998.89
Complications during procedure	998.11, 998.2, and 998.4
Specific complications of the gallbladder/digestive system	997.4, 576.0, 998.6, 576.4, 575.5, 51.36, 51.37, 51.39, 51.71, 51.72, and 51.79
Respiratory complications	997.3, 997.31, and 997.39
Other	998.4, 998.7, 998.8, 998.89, and 998.9

## Abbreviations

AAPC: Average annual percentage change
AC: Acute cholecystitis
AOR: Adjusted odds ratio
APC: Annual percentage change
CCI: Charlson Comorbidity Index
CI: Confidence interval
GP: General population
HLM: Hierarchical linear modeling
LIP: Low-income population
LOS: Length of hospital stay
MNDOP: Malignant neoplasm of digestive organs and peritoneum
NHI: National Health Insurance
NHIRD: National Health Insurance research database
ODGBT: Other disorders of gallbladder or biliary tract
SES: Socioeconomic status
